# Psychosocial Safety Climate Moderates the Effect of Demands of Hospital Accreditation on Healthcare Professionals: A Longitudinal Study

**DOI:** 10.3389/frhs.2022.824619

**Published:** 2022-04-22

**Authors:** Amna I. Alshamsi, Angeli Santos, Louise Thomson

**Affiliations:** ^1^Occupational Health-Psychology and Management, School of Medicine, University of Nottingham, Nottingham, United Kingdom; ^2^Applied Psychology, Centre for Organizational Health and Development, School of Medicine, University of Nottingham, Nottingham, United Kingdom; ^3^Occupational Psychology, Centre for Organizational Health and Development, School of Medicine, University of Nottingham, Nottingham, United Kingdom

**Keywords:** hospital accreditation, psychosocial safety climate, job demands-resources model, burnout, work engagement

## Abstract

Hospital accreditation has been studied comprehensively, yet few studies have observed its impacts on the burnout and work engagement levels of frontline healthcare professionals (HCPs). With a sample of 121 HCPs working in the United Arab Emirates' public hospitals, this study used a two-wave, cross-lagged panel design to examine the direct effects of job demands and job resources during hospital accreditations on burnout and work engagement and the moderating roles of psychosocial safety climate (PSC) on burnout and work engagement 3 months after accreditation. The data were analyzed using moderated structural equation modeling. As expected, we found that job demands (i.e., accreditation demands) had a direct effect on burnout, while job resources (i.e., social support) predicted work engagement. PSC moderated both relationships; however, it was not able to directly predict burnout or work engagement. Findings from this study show a positive relationship between accreditation demands and HCPs' health. Future research needs to examine the link between PSC and job demands-resources concepts before and after hospital accreditation more closely by using multiple time points to assess the causality relationships between predictor and outcome variables.

## Introduction

Workload and exhaustion have taken their toll on healthcare professionals (HCPs). It has become extremely difficult to ignore the pressures exerted on HCPs and their ever-growing levels of reported burnout. A recent review showed that HCPs experience high levels of burnout and work-related stress as a result of increasing demands and inadequate resources at work (1). Burnout, which is a syndrome characterized by feelings of emotional exhaustion and depersonalization (2), was found to affect 46% of doctors in the United States, one-third of physicians in the United Kingdom, and 12% of HCPs in Europe (3). Arab countries have shown comparable results for burnout among doctors. Abdulla et al. (4) conducted a cross-sectional study to estimate the prevalence of burnout among HCPs working in Qatar's healthcare facilities and found that 12.6% of doctors experienced burnout. Although many studies have provided evidence on some of the causes of burnout among HCPs, the roles of regulatory mechanisms and accreditation programs on HCPs' health and well-being have rarely been studied. Accordingly, the current study aims to examine the impact of job demands and resources faced by healthcare professionals during the period of external hospital accreditation.

Since the publication of “To Err is Human: Building a Safer Health System” by the Institute of Medicine (5), the IoM has been urging healthcare organizations to create safe environments for patients through regulatory mechanisms that include accreditation, licensing, and certification. As a result, many countries have employed hospital accreditations in order to develop environments that promote patient safety and healthcare quality. Accreditation assesses healthcare organizations based on their performance against a set of agreed standards. The assessment is carried out by external accrediting organizations to provide objectivity and external scrutiny (6). Healthcare facilities are therefore evaluated with regard to their structures and regulations on the quality of health services, including the availability of proper medical equipment, adequate staff ratios, and suitable quality measures (7). Although hospital accreditation has gained popularity in the last 20 years, there have been no detailed investigations into its impact on HCPs' working lives and their psychological health and wellbeing. Several studies have considered the unintended effects of hospital accreditation on staff, such as increased non-clinical workload, elevated stress levels prior to hospital accreditation, and reduced patient care (8, 9). Indeed, in a recent systematic review by Teoh et al. (10), HCPs' psychological health was found to mediate the relationship between working conditions—namely, job demands—and the quality of care provided to patients. Therefore, there is a salient and urgent need to investigate the impact and outcomes of hospital accreditation on staff in healthcare settings.

The present study seeks to contribute to the existing literature in three ways. First, it attempts to build on the findings of a qualitative study by Alshamsi et al. (11), which explored the psychosocial risks experienced by HCPs prior to hospital accreditation. This previous study found that HCPs experienced specific psychosocial demands prior to the hospital accreditation process, which included focusing on paperwork and attending additional training sessions or meetings. Such demands were perceived to influence HCPs' psychological health and the continuity of patients' care. Furthermore, Alshamsi et al. (11) found that HCPs sought social support, including perceived support from supervisors and colleagues, in order to reduce the effect of increased demands prior to accreditation. HCPs reported that teamwork and collaboration among individuals enhanced their engagement at work during the preparation phase of accreditation. The current study seeks to build on these qualitative findings by further examining the causal relationships between psychosocial working conditions and HCPs' psychological health and well-being during hospital accreditation in UAE hospitals. Specifically, it seeks to examine the relationships between job demands, job resources, and psychosocial safety climate (PSC) on burnout and work engagement of HCPs using a two-wave, longitudinal design. The third contribution of the study is to advance our understanding of the job demands-resources (JD-R) model through the inclusion of a new type of job demand that is specific to hospital accreditation and its interaction with PSC. Psychosocial safety climate is an innovative concept that combines work-related risks and safety climate, and it extends the current research through testing the role of PSC as an organizational resource in the JD-R model.

The job demands-resources model (12) is one of the most widely used and studied models in occupational health and psychology (13). The dynamic relationship between work characteristics and work outcomes has led to its wide acceptance, together with its flexibility in allowing researchers to incorporate job demands and resources that are unique to specific occupations into the model. According to the theory, job demands are specific characteristics of work that require employees to exert physical, social, or cognitive efforts, which could lead to physical or psychological impairments. Job resources, however, are job characteristics that help workers achieve their professional goals, reduce the effect of job demands, and stimulate professional growth through the learning and development of new skills (12, 14). It is well-established that increased job demands are significantly associated with higher levels of burnout, and that levels of burnout increase when job resources are insufficient, triggering what is known as the health impairment pathway. Such a pathway represents health problems that affect employees with high job demands, which may lead to the depletion of their energy and health (15, 16). Drawing on the concept of JD-R in healthcare services, Hu et al. (17) found that nurses with chronic job demands experienced high levels of burnout due to delayed recoveries from their persistently demanding jobs, which led to reductions in their energy levels in turn. Similarly, employees working in various healthcare occupations were found to experience exhaustion and cynicism when they were subjected to patient harassment and emotional demands (18).

Teoh et al. (10) were able to show an association between job demands and clinical excellence among healthcare workers in the UK, in which a negative relationship was observed between job demands and the quality of care provided to patients. In a more recent study, Teoh et al. (19), utilizing the framework of JD-R, found that job demands were more strongly associated with stress and presenteeism than job resources were with work engagement. Although these studies presented strong evidence on JD-R pathways, they relied on cross-sectional data. Therefore, causal relationships, as assumed by the JD-R model, would be better captured by a longitudinal design as employed in the present study and recommended by Rattrie and Kittler (13). Several studies have employed longitudinal designs to test the JD-R model in healthcare settings. For instance, a longitudinal study by Kraemer et al. (20) showed direct relationships between social stressors, time pressure, and the quality of care. Their findings suggested a relationship between job demands and the perceived quality of care irrespective of HCPs' psychological health and proposed that excessive job demands and adverse psychosocial work conditions can obstruct HCPs from working with patients effectively. Thus, the current study builds on the evidence to date to assess the significance of specific demands by using a bespoke construct to measure temporary job demands that arise during hospital accreditation (i.e., accreditation demands) and determine HCPs' psychological health and wellbeing after accreditation. We argue that in order to fully understand the impact of accreditation demands on burnout and strengthen the theoretical application of JD-R, there is an urgent need to consider the unique role of accreditation demands in the health impairment pathway.

Job resources explain the motivational process of the JD-R model through their relationship with work engagement. The current study seeks to assess the effect of one type of job resource, namely social support, on work engagement. Social support, which includes adequate support from supervisors and colleagues, occurs when people at work help or support their coworkers or subordinates as a form of assistance or coping mechanism (21). In the original conceptualization of JD-R, social support was tested as a job resource that reduced the effect of job demands in the health impairment pathway (15, 18). Furthermore, previous studies explored the direct effect of social support in predicting work engagement. Biggs et al. (22) suggested that working in an organization with a supportive culture can predict levels of support from supervisors and colleagues, which subsequently predict work engagement after 12–18 months. In a longitudinal study, Mauno et al. (23) investigated work engagement and its antecedents among Finnish healthcare workers, and they showed that job resources were robust predictors of the motivational pathway of the JD-R model. Therefore, the present study attempts to examine the role of social support from colleagues and supervisors during the process of hospital accreditation. Comprising of support from colleagues and supervisors, social support is operationalized as a job resource that is essential to increasing work engagement (16).

Psychosocial safety climate is an organizational construct intended to protect workers' psychological health and wellbeing through the presence of policies, procedures, and practices (24). As Dollard and Bakker [(24), p. 580] state, PSC is “freedom from psychological and social risk or harm.” The concept of PSC encapsulates how committed the management is to the employees' psychological health. PSC has extended research in physical safety climates (25) to include psychological risks, and it has been postulated as “the cause of the cause” of work-related stress and operationalized as a precursor to stressors found in workplaces (24). Nevertheless, several studies using longitudinal data have examined the role of PSC as a moderator in the JD-R model to predict employees' psychological health and positive organizational behavior (26–28). For instance, Dollard et al. (26) proposed that PSC enabled police officers to use organizational resources to reduce the effects of job demands on workers' psychological health. In their two-wave study, they found that the interaction between emotional demands and emotional resources was only evident when PSC scores were high. Moreover, they argued that utilizing job resources was contingent on levels of PSC, which suggests that organizations with high levels of PSC allow employees to utilize available job resources to reduce the effects of job demands.

PSC is considered an organizational-level construct by some researchers, and a considerable number of research studies have operationalized it at a group level (29). However, a recent review showed that PSC can also be operationalized at an individual level (30). Furthermore, PSC can be tested as a wider organizational resource. Dollard and Bakker (24), for instance, examined it as an organizational resource shaped by managers and supervisors. They operationalized PSC as an upstream workplace resource within the JD-R model and found that it had a strong moderating effect on burnout symptoms. Similarly, Hall et al. (27) adopted Dollard and Bakker (24) approach in examining PSC at the individual level to test its moderating effect on the relationship between job demands and depression. PSC is claimed to moderate different job demands because it allows individual workers to choose appropriate job resources to cope with work-related stress (27). We seek to extend the current research by testing the hypothesized buffering aspects of PSC as a wider hospital resource to determine whether it reduces the lagged effects of accreditation demands on personal burnout and boosts the lagged effects of social support on work engagement. The concept of work engagement in healthcare accreditation is necessary to connect HCPs with organizational values and goals, especially when improving the quality of care, to ensure successful and sustainable accreditation outcomes. We posit that the presence of adequate levels of PSC, prior to hospital accreditation and as perceived by individual HCPs, offers a safety net in terms of the available options that can help HCPs cope with demanding work situations and prevent the experience of burnout among them. As such, we predict that high scores on individual perceptions of PSC prior to hospital accreditation would buffer the effects of job demands on HCPs' psychological health and wellbeing and accentuate the relationship between job resources and positive motivational behaviors.

Furthermore, we argue that PSC ought to be operationalized at the individual level when used in conjunction with other individual-level, self-reported measures operationalized in this study. The current study attempts to examine relatively short-term demands, i.e., accreditation demands, which might affect some HCPs differently from other workers. Hence, we argue that there will be greater individual-level variability among HCPs in this context, which will influence their need to draw on job resources. In the current study, we propose that PSC is a macro-level workplace resource within the JD-R model, which allows individual employees to use available psychosocial resources when they experience high job demands.

### Aim and Hypotheses

The current study aims to contribute to this growing area of research through its novel examination of the roles of accreditation demands, social support, and psychosocial safety climate in predicting personal burnout and work engagement among healthcare professionals in the UAE during the period of hospital accreditation. Furthermore, we take a different analytical approach to testing the moderating effects of PSC by modeling all the relationships of the JD-R model and their interactions. While previous studies have provided evidence on the moderating effect of PSC within the JD-R model, the majority have tested either health impairment or motivational paths separately and have rarely included both paths in one model. However, our study attempts to test both paths in a single, more parsimonious model that combines job demands and job resources constructs. The study also aims to test PSC interactions with the accreditation demands and social support variables in predicting burnout and work engagement among HCPs.

**Figure 1:**
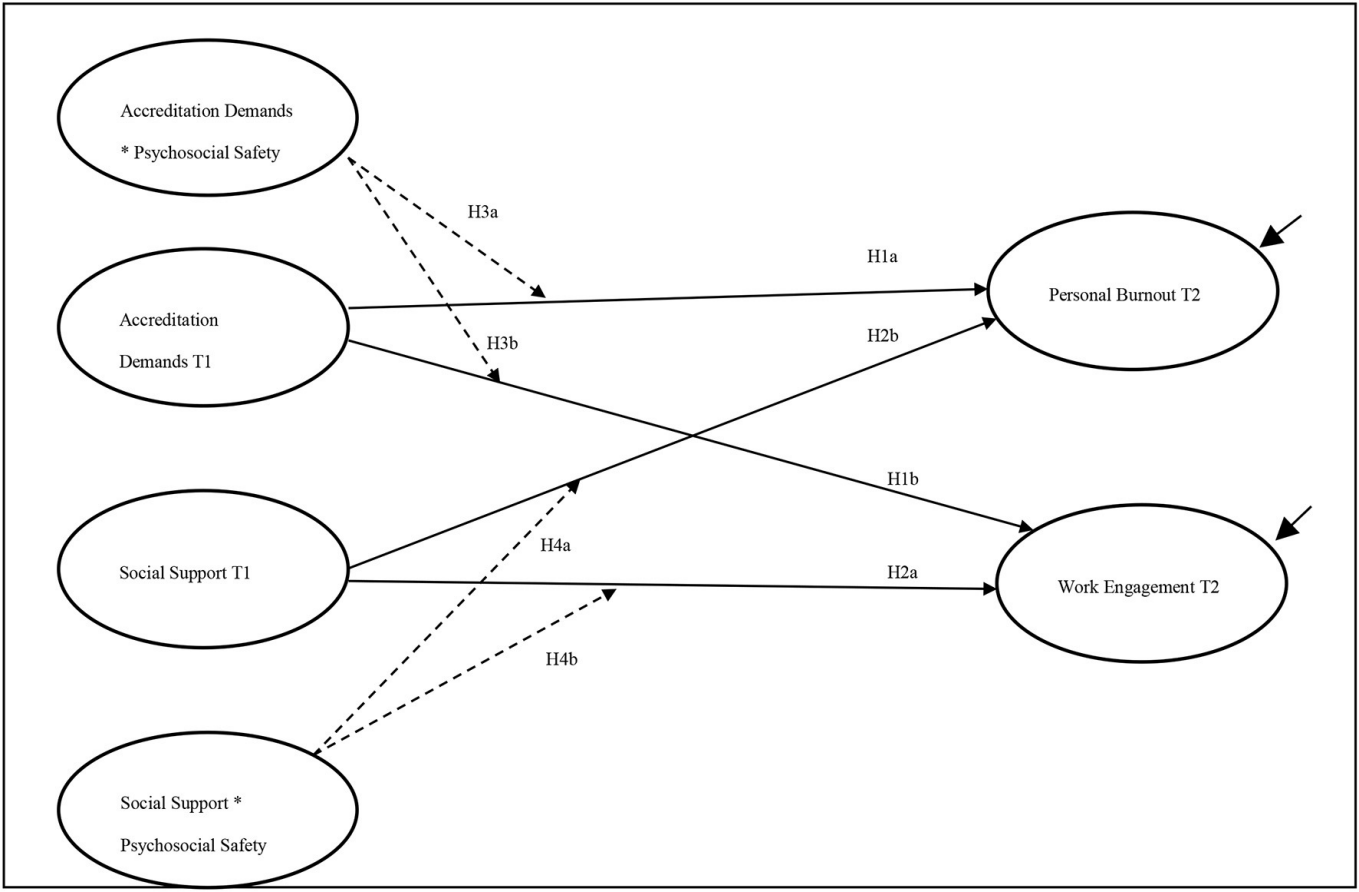
Research model and hypotheses. Paths are estimated as a lagged paths from Time 1 to Time 2. Black solid lines represent direct effect; dashed lines represent interactions.

Few empirical studies have tested the JD-R model and the PSC construct in the Middle Eastern region. Therefore, another contribution of the current study is to test the moderating role of PSC using structural equation modeling (SEM) at an individual level. What this research does differently is it attempts to test the developed construct and incorporate it into the JD-R model using SEM analysis techniques. This approach enables the progression of a theoretical insight that is useful in hospital accreditation, where existing models are absent or incomplete. Consequently, this study attempts to test the following hypotheses (Figure 1):

Hypothesis 1: Accreditation demands are positively related to personal burnout—that is, the health impairment path (H1a)—and negatively related to the work engagement and motivational paths (H1b).
Hypothesis 2: Social support is positively related to work engagement (H2a) and negatively related to personal burnout (H2b).
Hypothesis 3: Psychosocial safety climate interacts with accreditation demands to moderate the health impairment path (H3a) and motivational path (H3b).
Hypothesis 4: Psychosocial safety climate interacts with social support to moderate the health impairment path (H4a) and motivational path (H4b).
Hypothesis 5: Adding interaction paths will improve the fit of the model.

## Methods

The study was conducted from September 2019 to March 2020 with employees working in two public hospitals in the UAE, and data collection was ceased days before the announcement of a national lockdown due to the COVID-19 pandemic. HCPs working in government hospitals regulated by the Ministry of Health and Prevention (MOHAP) took part in the study. These hospitals were selected based on their accreditation status, as inspection visits to assess the quality of their services were scheduled within 3 months from the start of the study. Ethics approvals were granted by the MOHAP (MOHP/DXB-SU BC-No-40/2018) and University of Nottingham's Division of Psychiatry and Applied Psychology (DPAP) ethics committees (i.e., Reference Number 0412).

### Study Population

The data were collected at two time points, 6 months apart: 3 months before the accreditation inspectors' visit (T1) and 3 months after their visit (T2). A 6-month time-lag was used to measure the effects of job demands and job resources on HCPs' burnout and work engagement levels directly after accreditation. Data were collected through self-completion online surveys. Invitations to participate in the study were sent to all HCPs working in the two hospitals (*n* = 911) and had a role in the accreditation process. The total response rate was 289 (31.7%) in T1 and 220 (24.1%) in T2. Participants who had responded to both T1 and T2 surveys were identified using a unique coding system, providing a final sample of 121 matched participants across the two hospitals. These codes were developed according to the demographic questions presented in the questionnaire, which include participants' gender, year of birth, and last three digits of participants' file number. Of the 121 matched responses, 68.6% (*n* = 83) were from the first hospital and 31.4% (*n* = 38) were from the second hospital.

All participants were above the age of 25 years, and the average age of the respondents was 39.25 years (SD = 7.6), with 45.5% of the participants aged between 36 and 45 years. Of the total participants, 80.2% (*n* = 97) were female and 19.8% (*n* = 24) were male. An unequal number of responses between males and females in the healthcare sector is expected, as more females than males tend to occupy nursing jobs. The sample consisted of doctors (7.4%), nurses (85.1%), allied health professionals (1.7%), administrative workers (2.5%), and workers from other professions (3.3%). In addition, 47.1% of participants had a clinical role, and 46.3% had both clinical and administrative roles. Measuring the roles of the participants was important, as HCPs might experience conflicting needs to balance between their role of caring for patients and their role of preparing for hospital accreditation.

The study selected the participants with matching codes to continue with the cross-lagged moderation analysis. Therefore, independent sample *t*-tests were performed at T1 to check for systematic bias between participants with matching codes (*n* = 121) and participants who only completed one survey (*n* = 168) and had been excluded from the final sample. All variables were included in the independent sample *t*-test analysis to minimize differences between both groups. The results showed a non-significant difference in the accreditation demands variable [*t*(287) = 0.24, *ns*]; social support [*t*(276) = 0.30, *ns*]; PSC-4 [*t*(271) = 0.10, *ns*]; personal burnout [*t*(269) = −1.4, *ns*]; and work engagement [*t*(271) = 0.20, *ns*]. Similar results were found in T2 when compared with included and excluded cases. Consequently, we dismissed any indication of selection bias among the participants.

### Measures

Survey items were presented in the two main official languages of UAE, Arabic and English. Since some of the measurement tools had not been translated to Arabic, we adopted a forward and back translation technique (31) to convert the questionnaire items into Arabic to increase the response rate.

#### Accreditation Demands (T1 and T2)

In this study, we tested the accreditation demands construct, which was formulated and added to measure the unique demands of hospital accreditation. These items reflected the findings from a qualitative study that aimed to explore the psychosocial risks experienced by HCPs due to hospital accreditation (11). Four items of the accreditation demands construct were measured using a five-point Likert scale ranging from 1 (always) to 5 (never or hardly ever), and a score of 0 was assigned to the option of “not applicable.” These items are as following: (1) “Do you feel that your current work takes so much from your time that you cannot treat patients properly,” (2) “Do you feel that the required paperwork you are expected to complete keeps you away from patients?,” (3) “Do you feel the documentation requirements keeps you away from patients?,” and (4) “Do you feel that additional activities such as attending training or meetings keeps you away from patients?” The reliability of the accreditation demand scale was 0.79.

#### Job Resources (T1 and T2)

The job resource construct was measured using six items from the COPSOQ II scale (32). This construct involved three items from *Social Community at Work* and three items from *Social Support Superior*, which were examined using a five-point Likert scale that ranged from a high score of 100 (Always) to a low score of 0 (never or hardly ever). The reliability of the social support scale was 0.91.

#### Psychosocial Safety Climate (T1 and T2)

The current study used the four items of PSC-4 derived from PSC-12 (33). Responses are made on a five-point Likert scale ranging from 5 (strongly agree) to 1 (strongly disagree). Berthelsen et al. (34) have established a benchmark to identify levels of PSC-4 in organizations. We followed their recommended benchmark, which considers a score >12 to be a good level, while a score below 8 is a poor level. A moderate level will have a score between 12 and 8 (34). The reliability of the scale was 0.93.

#### Burnout (T1 and T2)

Burnout was measured using the Copenhagen Burnout Inventory (35). The study included a personal burnout construct (six items). Personal burnout aimed to measure the general psychological exhaustion experienced by individuals without a specific cause. Items were measured using a five-point Likert scale ranging from a high score of 100 (always) to a low score of 0 (never or hardly ever). The reliability of the personal burnout scale was 0.92.

#### Work Engagement (T1 and T2)

Work engagement, which refers to the high energy employees displayed toward their workplaces, was measured using the shortened version of the Utrecht Work Engagement Scale (UWES) (36). This version of the UWES had nine items and is scored on a seven-point Likert scale ranging from 0 (never) to 6 (always). However, only seven items were included in the study due to low factor loadings for Items 1 and 2. The reliability of the work engagement scale (seven items) was 0.93.

#### Control Variables (T1)

The study controlled for two variables: age (numeric variable) and clinical role (1 = clinical role only, 0 = other roles). These variables were reported in T1 and T2 and considered as stable variables; hence, they were included as T1 variables only in the analysis.

### Statistical Analysis

MPlus version 8.4 (37) was used to analyze the data using the maximum likelihood estimator to evaluate the covariance matrix. A confirmatory Factor Analysis (CFA) was performed to the items of the variables included in the model at T1, such as: Accreditation Demands, Social Support, PSC-4, Personal Burnout, and Work Engagement. The results of the CFA demonstrated an acceptable fit, chi-square (312) = 484.1, *p* < 0.05, CFI = 0.950, RMSEA = 0.056, and SRMR = 0.071. Observed factors were presented as manifest variables that were included in Time 1 (T1) and Time 2 (T2).

Structural Equation Modeling (SEM) and cross-lagged panel design were applied to test the hypotheses for the moderating role of PSC in the JD-R model. PSC was measured at the individual level representing a wider organizational resource. Because of the multilevel nature of PSC implied in the literature, and the small sample size, a vital step was taken to assess whether the data can be aggregated at the hospital level. The intraclass correlation coefficient (ICC) was calculated for every dependent variable at T2, that is, Personal Burnout and Work Engagement. It is recommended that the statistical independence of groups should be ignored if the attributed variance is <5% (38). The ICC showed that 0.1% of the variance in personal burnout and 0% of variance in work engagement might be attributed to hospital level. Thus, observations were treated as statistically independent.

Total mean standardized scores were utilized in subsequent statistical analysis for Accreditation Demands, Social Support, Personal Burnout, and Work Engagement. A total score for PSC-4, ranging from to 4 to 20, was obtained to assess benchmark levels of PSC-4 (34). To perform SEM, direct paths were tested between independent variables, that is, Accreditation Demands and Social Support, and PSC-4 and outcome variables, that is, Personal Burnout and Work Engagement. PSC-4 was included as a moderator of health impairment and motivational paths. To test the moderating effect of PSC, two interaction variables were created (39). The first interaction was the product of multiplying accreditation demands by PSC-4. The second interaction was the product of multiplying social support by PSC-4. To test the model, all variables were standardized to account for the different scoring techniques and were allowed to be correlated to account for the assumptions of cross-lagged models (38). The significant interactions were plotted using the techniques and Microsoft Excel sheet proposed by Dawson and Richter (40).

Two models were tested and compared. Model 0 (M0) is a simpler model, which includes direct paths from predictor variables to outcome variables. Model 1 (M1) included paths similar to M0 and the two direct paths from the interaction products between Accreditation Demands and PSC-4, and Social Support and PSC-4. To assess the moderating effects, the paths from the interaction coefficients to outcome variables should be statistically significant. Furthermore, the tested model (M1) needs to show a better fit when compared to the simpler model (M0). Such an approach is considered effective for testing the differences in Chi-square (χ^2^) in different competing models. For an adequate fit, we followed Hu and Bentler (41) 17 to present a combination of fit indices where SRMR <0.08, RMSEA <0.06, CFI >0.95, and a minimum value of AIC, log-likelihood, and χ^2^.

## Results

Table 1 presents the means, standard deviations, reliability of the measures, and Pearson's correlation matrix to improve our understanding of the changes in the constructs over time. The mean score for PSC-4 was 12.6 for T1 and 13.6 for T2, which suggested a good level of PSC [Berthelsen et al., (17)]. The internal consistencies of the measures were acceptable with Cronbach's alpha > 0.75, except for accreditation demands' measures, which showed alpha values of 0.64 and 0.68 in T1 and T2, respectively. While some authors suggest that a Cronbach value between 0.7 and 0.6 indicates a moderate to acceptable value (42), we decided to keep the accreditation demands construct, since it was formulated for this study and had never been measured before. The Pearson's correlation matrix showed consistent results with those of previous studies. In T1, PSC-4 was negatively related to T2 personal burnout and positively related to work engagement. The accreditation demands construct was positively related to personal burnout after accreditation. Social support, on the other hand, was positively related to work engagement and negatively related to personal burnout. Finally, both interaction products were negatively related to personal burnout and positively related to work engagement.

**Table 1:** Pearson's correlation among variables with means, standard deviations (SD), and internal consistencies (*n* = 121).

	**Variables**	**Mean**	**S.D**.	**1**	**2**	**3**	**4**	**5**	**6**	**7**	**8**	**9**	**10**	**11**	**12**	**13**	**14**	**15**	**16**
1	Age	39.18	7.6	1															
2	Clinical role	0.78	0.5	−0.08	1														
3	Accreditation demands T1	2.65	1.2	−0.01	0.05	(0.64)													
4	Social support T1	78.40	20.5	−0.14	−0.06	0.14	(0.94)												
5	Psychosocial safety climate T1	12.68	3.9	−0.04	0.03	0.14	0.21^*^	(0.93)											
6	PSC-4 * accreditation demands T1	0	1	−0.05	−0.003	0.82^**^	0.20^*^	0.61^**^	(-)										
7	PSC-4 * social support T1	0	1	−0.11	−0.04	0.18^*^	0.67^**^	0.84^**^	0.57^**^	(-)									
8	Personal burnout T1	46.32	20.2	−0.07	−0.02	0.01	−0.37^**^	−0.28^**^	−0.14	−0.39^**^	(0.91)								
9	Work engagement T1	4.54	1.2	0.08	−0.09	0.10	0.41^**^	0.17	0.08	0.33^**^	−0.27^**^	(0.93)							
10	Accreditation demands T2	2.64	1.2	0.09	0.17	0.24^**^	0.03	0.17	0.20^*^	0.14	0.07	0.13	(0.68)						
11	Social support T2	76.67	20.7	−0.05	0.05	0.02	0.45^**^	0.12	0.05	0.29^**^	−0.24^**^	0.11	−0.16	(0.93)					
12	Psychosocial safety climate T2	13.67	4.0	−0.03	0.01	0.01	0.14	0.37^**^	0.13	0.31^**^	−0.11	0.21^*^	0.08	0.19^*^	(0.97)				
13	PSC-4 * accreditation demands T2	0	1	0.03	0.13	0.16	0.12	0.27^**^	0.20^*^	0.26^**^	−0.03	0.17	0.86^**^	−0.03	0.51^**^	(-)			
14	PSC-4 * social support T2	0	1	−0.04	0.04	−0.02	0.33^**^	0.30^**^	0.08	0.36^**^	−0.21^*^	0.22^*^	−0.04	0.74^**^	0.78^**^	0.31^**^	(-)		
15	Personal burnout T2	42.94	22.3	−0.21^*^	0.14	0.07	−0.18	−0.13	−0.04	−0.18	0.41^**^	−0.23^*^	0.02	−0.33^**^	−0.43^**^	−0.19^*^	−0.48^**^	(0.93)	
16	Work engagement T2	4.48	1.2	0.08	−0.15	0.18^*^	0.28^**^	0.05	0.16	0.14	−0.23^*^	0.37^**^	−0.08	0.55^**^	0.11	−0.02	0.39^**^	−0.50^**^	(0.93)

* *p < 0.05 (2-tailed)*,

** *p < 0.01 (2-tailed)*.

Pairwise *t*-tests were performed to assess the mean differences of variables across time, i.e., T1 and T2. The average score of accreditation demands hardly decreased and showed a non-significant mean difference between T1 and T2 [*t*(120) = −0.004, *ns*]. Likewise, social support did not change significantly [*t*(117) = −1.6, *ns*]; however, the mean differences showed a negative value, which indicated a reduction in perceived social support. Personal burnout and work engagement also showed non-significant changes over time: *t*(116) = −3.8, *ns* and *t*(115) = −0.05, *ns*, respectively. Both personal burnout and work engagement mean values decreased after accreditation. Finally, PSC-4 significantly increased between T1 and T2 [*t*(116) = 1.0, *p* < 0.05].

### Main Effects

The stability of the constructs from T1 to T2 was assessed using an a-priori model that included only autoregression paths, which were significantly not equal to zero. The autoregressive coefficients included accreditation demands (*b* = 0.36, *p* < 0.01); social support (*b* = 0.35, *p* < 0.01); PSC-4 (*b* = 0.41, *p* < 0.01); personal burnout (*b* = 0.41, *p* < 0.01); and work engagement (*b* = 0.37, *p* < 0.01). The interaction coefficients also showed significant autoregressive paths for accreditation demands and PSC-4 (*b* = 0.33, *p* < 0.01), and social support and PSC-4 (*b* = 0.35, *p* < 0.01). Both models included paths of the a-priori model and cross-lagged paths between variables in T1 and T2. Furthermore, these models were adjusted for the participants' ages and clinical roles. Model fits are presented in Table 2, which compares M0 to M1 using the minimum values of AIC, LL, and χ^2^. M1, which included moderation effects, indicated a good fit when compared with M0 and had a minimum value of −2LL and AIC: *X*^2^ (33) = 41.37, *ns*, CFI = 0.991, RMSEA = 0.048, and SRMR = 0.062.

**Table 2:** Fit statistics for the study models (*n* = 121).

**Model**	**X^**2**^**	**df**	***P*-value**	**CFI**	**RMSEA**	**SRMR**	**-2LL**	**AIC**	**Model comparisons**	**Δ *X*^2^**	**Δ df**	**Δ-2LL**
M0	13.31	14	0.502	1.000	0.000	0.046	1530.716	3143.43				
M1	41.37	33	0.150	0.991	0.048	0.062	1456.582	3043.17	M0 vs M1	28.06	19	148.27^**^

*df, Degree of Freedom; CFI, Comparative fit Indices; RMSEA, Root Mean Square Error of Approximation; SRMR, Standardized Root Mean Square Residual; AIC, Akaike Information Criterion; LL, Log-Likelihood; Δ, differences in estimations; X^2^, Chi-Square*.

^*^p < 0.05 (2-tailed),

** *p < 0.01 (2-tailed)*.

Table 3 shows that the accreditation demands variable was positively related to personal burnout and negatively related to work engagement in the two models (M0 and M1). Furthermore, the social support variable was negatively related to personal burnout and positively related to work engagement. However, these results were not statistically significant. After controlling for age and clinical role, there was a statistically significant direct relationship between accreditation demands T1 and personal burnout T2 (*b* = 0.54, *p* < 0.01). Furthermore, a significant direct effect was found between social support T1 and work engagement T2 (*b* = 0.47, *p* < 0.05) after controlling for the clinical role only. These results support H1a and H2a. Although the clinical role variable had a relatively small effect on personal burnout T2 (*b* = 0.17, *p* < 0.05) and work engagement T2 (*b* = −0.19, *p* < 0.01), such effects were statistically significant. Age was negatively related to personal burnout T2 (*b* = −0.16, *p* < 0.05), which indicated that HCPs experience less burnout with age.

**Table 3:** Results of the moderated structural equation models: interactions of Accreditation Demands and Social Support with PSC-4.

**Model**	**Predictors**	**Personal Burnout at Time 2**			**Work Engagement Time 2**		
		**β**	**b**	**95% CI**	**β**	**b**	**95% CI**
Model 0	Accreditation demands T1	1.02	0.05	(-0.092, 0.199)	0.20	0.20^**^	(0.055, 0.334)
	Social Support T1	−0.06	−0.06	(-0.22, 0.107)	0.01	0.15	(-0.033, 0.332)
	Psychosocial Safety Climate T1	−0.25	−0.04	(-0.21, 0.126)	−0.02	−0.07	(-0.213, 0.075)
	Age	−0.58	−0.19^**^	(-0.338,−0.05)	0.01	0.06	(-0.078, 0.204)
	Clinical Role	8.47	0.19^*^	(0.044, 0.332)	−0.47	−0.19^**^	(-0.328,−0.05)
Model 1	Accreditation demands T1	10.50	0.54^**^	(0.162, 0.925)	−0.20	−0.19	(-0.604, 0.218)
	Social support T1	−0.23	−0.21	(-0.598, 0.186)	0.03	0.47^*^	(0.053, 0.877)
	Psychosocial safety climate T1	0.40	0.07	(-0.482, 0.617)	0.07	0.23	(-0.332, 0.793)
	PSC-4 * accreditation demands T1	−14.70	−0.64^*^	(-1.121,−0.161)	0.60	0.49	(-0.029, 1.011)
	PSC-4 * social support T1	5.79	0.25	(-0.442, 0.948)	−0.92	−0.75^*^	(-1.478,−0.023)
	Age	−0.48	−0.16^*^	(-0.284,−0.035)	-	-	(-,-)
	Clinical role	7.78	0.17^*^	(0.036, 0.303)	−0.46	−0.19^**^	(-0.327,−0.05)

*PSC-4, psychosocial safety climate 4 items; β, unstandardized path coefficients; b, standardized path coefficients*.

* *p < 0.05 (2-tailed)*,

** *p < 0.01 (2-tailed)*.

### Interaction Effects

**Figure 2:**
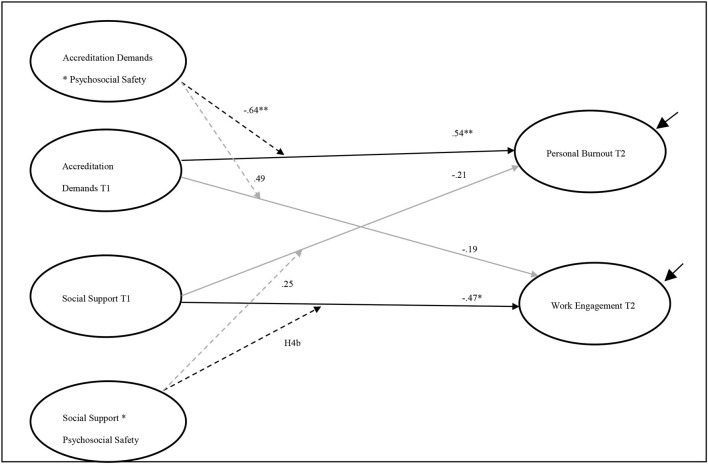
The final model including direct and indirect paths. Black solid lines represent direct effect; dashed lines represent interactions; gray lines represent insignificant effect. PSC, Psychosocial Safety Climate. χ^2^ (33) = 41.37, *p* = ns, CFI = 0.991, RMSEA = 0.048, and SRMR = 0.062. The symbol * indicates the values of *P* < 0.005 and the symbol ** indicates the values of *P* < 0.001.

**Figure 3:**
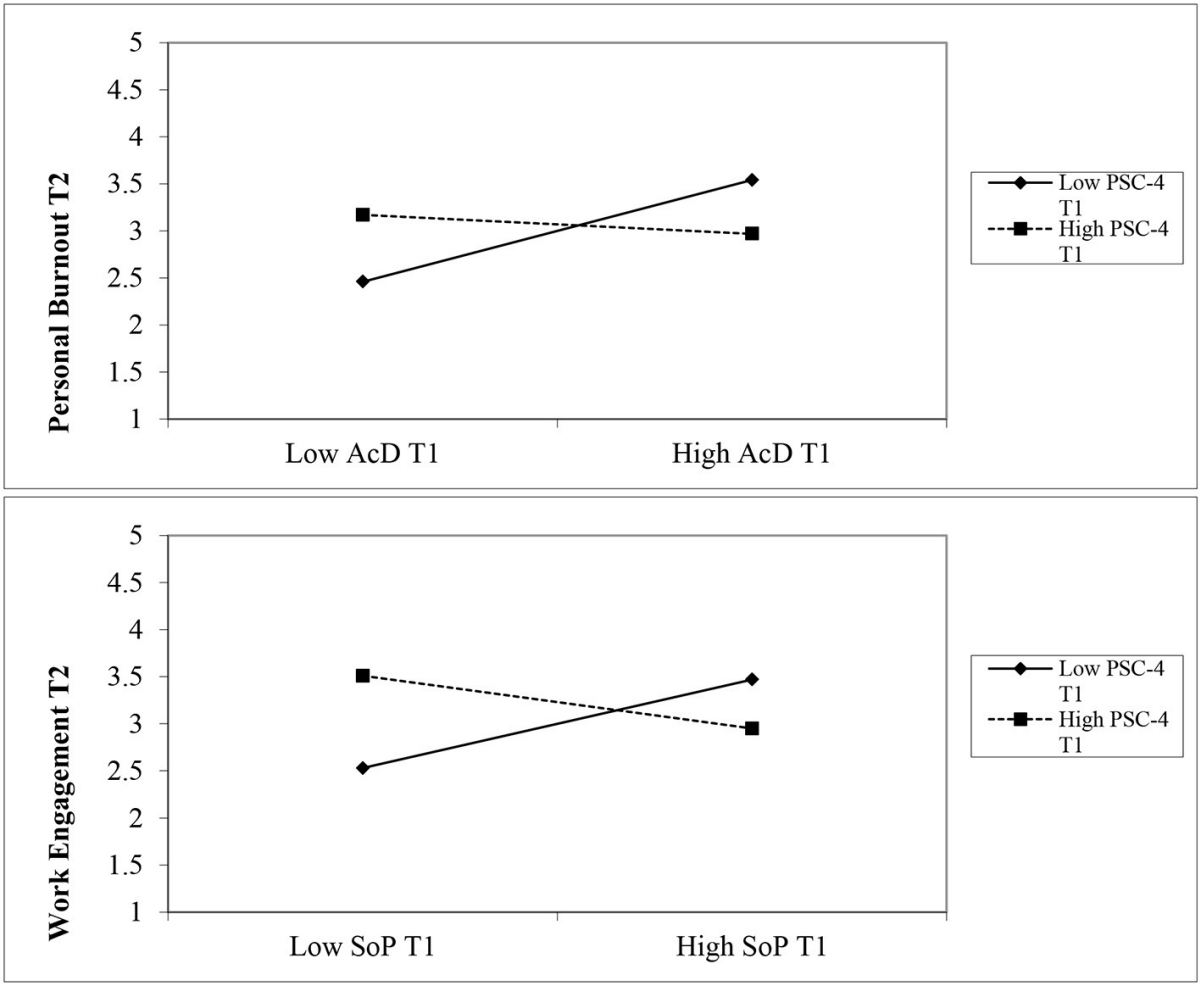
Upper plot: a significant interaction effect of PSC-4 on the relationship between Accreditation Demands and Personal Burnout. Lower plot: a significant interaction effect of PSC-4 on the relationship between Social Support and Work Engagement. Note: AcD: Accreditation demands; SoP: Social Support; PSC-4: Psychosocial Safety Climate; T1: Time 1; T2: Time 2.

Figure 2 presents the paths from the final model. Of the four possible moderation effects of PSC-4 on the outcome variables, only two interactions had lagged effects on personal burnout and work engagement in T2. As shown in Table 3, the result of the interaction between accreditation demands and PSC-4 supports H3a. A significant negative correlation was found between this interaction and personal burnout T2 (*b* = −0.64, *p* < 0.01). Although the interaction between accreditation demands and PSC-4 had a positive relationship with work engagement, the path was not significant (*b* = 0.49, *ns*). On the other hand, the interaction between social support and PSC-4 showed a statistically significant effect on work engagement T2 (*b* = −0.75, *p* < 0.05), supporting H3b; however, the effect was in the opposite direction (Figure 3). PSC-4 T1, on the other hand, showed a non-significant interaction between social support and moderate personal burnout T2 (*b* = 0.25, *ns*).

The direct paths from PSC-4 in T1 to the outcome variables—personal burnout and work engagement—were not significant: *b* = 0.10, *ns* and *b* = 0.23, *ns*, respectively. It is worth noting that the lagged direct effects of PSC-4 on the outcome variables were smaller than the lagged effects of the interaction effects, suggesting greater importance for the moderating effect of PSC-4 than its direct effect. Furthermore, the addition of interaction paths improved the fit of the model. The moderation M1 model showed a better fit when compared with M0, which contained the direct effects only. The estimation of the LL deviance of M1 significantly decreased when compared with M0 [Δ −2LL (19) = 148.3, *p* < 0.01]. The AIC in M1 had the minimum value, compared with M0. These estimates indicated that M1, with the interaction paths, had a better fit for the data, which supports H5.

## Discussion

This study introduced accreditation demands—a novel type of job demand specific to the accreditation process—and examined their effects on HCPs' levels of burnout. This overarching aim was tested using two psychological frameworks: the JD-R model and the PSC concept. Both were tested within a unique and transitory context—that of hospital accreditation. The current study used a short version of the PSC scale to test its potentially moderating effect in line with the JD-R model and in relation to HCPs' psychological health (i.e., personal burnout) and positive organizational behavior (i.e., work engagement) after the accreditation inspection.

We found that the direct effect of accreditation demands in T1 predicted personal burnout in T2. Further, job resources in T1, that is, social support, was found to have a positive impact on work engagement in T2. These findings broadly support the work of other studies in this area with the JD-R framework (17, 22). Furthermore, participants with a clinical role in T1 experienced higher levels of burnout and lower levels of work engagement in T2. Such findings might be explained by the conflict demands of accreditation exerted on HCPs and their role to maintain good quality of patients' care, which broadly supports the work of other studies in the area of examining burnout among HCPs (43).

We found that PSC moderated the unique aspects of job demands during accreditation. Levels of PSC prior to the accreditation visit were high (mean = 12.6); nevertheless, the findings demonstrate that perceived PSC can buffer the relationship between accreditation demands and negative psychological health outcomes. However, the results of PSC interaction with social support did not enhance HCPs positive organizational behavior. These results support the findings of previous studies for the moderating role of PSC in both the health impairment (24, 26–28). In the present study, PSC acted as an organizational resource for HCPs to utilize to mitigate the negative effects of accreditation demands. The results of the current study confirm the importance of PSC as a moderator, over and above any direct effects.

The theoretical contribution of the current study is the investigation of PSC, as an organizational resource, and the application of JD-R model in a Middle Eastern context. The advancement of such theoretical models was achieved through the addition of a new construct that is unique to hospital accreditation. This study aligns with evidence from previous observations that confirm the moderating role of PSC within the JD-R model (24). The impact of job demands on burnout is well-established (12, 44); however, there is a growing body of literature that recognizes the importance of PSC in moderating such an impact in certain transitory settings, such as that of hospital accreditation. These findings raise intriguing questions regarding the nature and extent of the unintended consequences of hospital accreditation on HCPs' health and wellbeing. Therefore, the concept of PSC provides a practical measurement tool to assess working conditions that explain job demands, job resources, and their outcomes. Future research should focus on the work environment and on supporting hospital systems prior to accreditation to mitigate the impact of such processes on frontline HCPs. Further, managers and policy makers need to acknowledge such an impact by developing robust legislation that aims for healthy HCPs and continuity of patients' safety.

Despite these findings, this study has some limitations. The most important limitation lies in the fact that the current study collected data at the individual level rather than the organizational level. PSC was originally theorized to be a distinct feature of organizations; therefore, studies in this field tended to aggregate data in order to understand the relationship between the organizational climate and relevant outcomes (45). However, this study assessed PSC at the individual level as a result of the limited number of public hospitals that had gone through accreditation during the data collection period. Moreover, the analyses of intraclass coefficients revealed almost no variation when grouping variables according to hospital level, and the observations were treated as statistically independent. Nevertheless, we used four items of the existing PSC-12 instrument (33, 34), previously tested at an individual level, and the psychometric properties of the scale did not change. Aside from that, having mostly females and nurses as respondents might have contributed to gender imbalance and imposed a further limitation.

Due to the pandemic, it was difficult to recruit more hospitals or conduct a three-wave panel study. It was essential to stop collecting data in March 2020, which limited the sample size. A small sample size might have affected the statistical power of the findings, so they cannot be conclusive or generalized. However, the results of this paper were in line with previous research. Also, it can be argued that the JD-R model cannot be falsified, since demands and resources vary among organizations (46). Future research should aim for data to be collected at multiple time points to enhance the understanding of the possible effects of hospital accreditation on work health and organizational behavior and whether such effects fade away with time. Regardless, most research on PSC has been carried out in two-wave longitudinal designs and supports its moderating role on work conditions (26).

Furthermore, the lagged direct effects of PSC on the outcome variables were non-significant compared with the lagged effects of its interaction effects. According to Field (47), a significant interaction effect does not justify a causal effect. Therefore, the role of PSC on HCPs' health should be interpreted with caution. The final limitation of the study is the use of a self-report questionnaire. Despite its limitations, the study adds to our understanding of the buffering aspects of PSC on the effect of the JD-R model attributed to the process of quality inspection programs. In addition, the study was able to examine the role of PSC within the JD-R framework in a Middle Eastern context, that of the UAE, thus responding to the calls for much-needed research in the field of occupational health and psychology in non-Western contexts.

## Data Availability Statement

The raw data supporting the conclusions of this article will be made available by the authors, without undue reservation.

## Ethics Statement

The studies involving human participants were reviewed and approved by MOHAP (MOHP/DXB-SU BC-No-40/2018) and the University of Nottingham's Division of Psychiatry and Applied Psychology (DPAP) Ethics Committees (i.e., Reference Number - 0412). The patients/participants provided their written informed consent to participate in this study.

## Author Contributions

AA contributed to the design of the study, the collection of data, the analysis of data, and the writing of this manuscript. AS contributed to the analysis of the data and the preparation of the manuscript. LT read and critically revised the manuscript. All authors read and approved the final manuscript.

## Funding

This study is part of a Ph.D. project on the effect of hospital accreditation on frontline healthcare professionals' psychological health. This research was self-funded, although the Ministry of Education of the United Arab Emirates has provided a grant for the corresponding author's tuition fees.

## Conflict of Interest

The authors declare that the research was conducted in the absence of any commercial or financial relationships that could be construed as a potential conflict of interest.

## Publisher's Note

All claims expressed in this article are solely those of the authors and do not necessarily represent those of their affiliated organizations, or those of the publisher, the editors and the reviewers. Any product that may be evaluated in this article, or claim that may be made by its manufacturer, is not guaranteed or endorsed by the publisher.

## Abbreviations

HCPs: Healthcare Professionals
UAE: United Arab of Emirates
JD-R: Job Demands-Resources model
PSC: Psychosocial Safety Climate
COPSOQ II: The second version of the Copenhagen Psychosocial Questionnaire
UWES: Utrecht Work Engagement Scale
SRMR: Standardized Root Mean Squared Residual
RMSEA: Root mean square error of approximation
CFI: Comparative fit index
AIC: Akaike Information Criterion.
